# Critical Thinking: The ARDESOS-DIAPROVE Program in Dialogue with the Inference to the Best and Only Explanation

**DOI:** 10.3390/jintelligence11120226

**Published:** 2023-12-16

**Authors:** Miguel H. Guamanga, Fabián A. González, Carlos Saiz, Silvia F. Rivas

**Affiliations:** 1 Faculty of Human Sciences, Universidad Icesi, Cali 760000, Colombia; miguel.guamanga@u.icesi.edu.co; 2 Faculty of Science and Education, Politécnico Colombiano Jaime Isaza Cadavid, Medellín 050021, Colombia; fabiangonzalez@elpoli.edu.co; 3 Department of Basic Psychology, Psychobiology and Methodology of Behavioral Sciences, University of Salamanca, 37005 Salamanca, Spain; csaiz@usal.es

**Keywords:** critical thinking, ARDESOS-DIAPROVE program, epistemology, explanation, inference to the best explanation

## Abstract

In our daily lives, we are often faced with the need to explain various phenomena, but we do not always select the most accurate explanation. For example, let us consider a “toxic” relationship with physical and psychological abuse, where one of the partners is reluctant to end it. Explanations for this situation can range from emotional or economic dependency to irrational hypotheses such as witchcraft. Surprisingly, some people may turn to the latter explanation and consequently seek ineffective solutions, such as visiting a witch doctor instead of a psychologist. This choice of an inappropriate explanation can lead to actions that are not only ineffective but potentially harmful. This example underscores the importance of inference to the best explanation (IBE) in everyday decision making. IBE involves selecting the hypothesis that would best explain the available body of data or evidence, a process that is crucial to making sound decisions but is also vulnerable to bias and errors of judgment. Within this context, the purpose of our article is to explore how the IBE process and the selection of appropriate explanations impact decision making and problem solving in real life. To this end, we systematically analyze the role of IBE in the ARDESOS-DIAPROVE program, evaluating how this approach can enhance the teaching and practice of critical thinking.

## 1. Introduction

The purpose of an educational intervention is intrinsically causal (Ennis 1973). Its goal is to induce changes in the student through a series of conceptual, methodological, pedagogical, and didactic proposals that translate into expected results in academic performance and student behavior (Anderson and Krathwohl 2001). The teaching and learning of critical thinking (CT) cannot be separated from this causal notion. The key questions of any CT program are as follows: what learning objectives should guide the intervention? What content should be taught? Which strategies should be implemented? And self-critically, why do it, and what changes are expected?

Through the literature on CT, essential skills, dispositions, and knowledge can be identified that define it and are reflected in the curricular designs of direct instruction (Fasko 2003; Saiz 2017). The timeline that Thomas and Lok (2015) trace from Dewey to the present day with theoretical works and practical manuals on CT shows that despite the lack of consensus on a defined concept of CT, the associated skills are a common meeting point. The classic references of CT have highlighted interpretation, explanation, analysis, inference, evaluation, and self-regulation (Ennis 2015; Facione 1990; Halpern and Dunn 2023) with varying degrees of intensity. The same has happened with dispositions such as being analytical, systematic, inquisitive, having an open mind, being willing to seek the truth and the precision that the situation requires (Ennis 2015). Necessary knowledge has also been identified, such as general knowledge, professional expertise, and knowledge in formal processes of logic, mathematics, and statistics (Glaser 1942).

Understanding this conceptual framework invites, first of all, to think that the task of training in critical thinking is infinite, especially considering that the real world has not yet appeared as the protagonist of the conceptual conglomerate. Secondly, it invites us to value the valuable accumulated knowledge that we have today about CT from philosophy, cognitive psychology, and critical pedagogy (Jahn and Kenner 2018).

In this timeline, it can be traced how some skills have been prioritized, which often undermines other skills or leaves them forgotten in instructional designs. It can be observed that the discourse of CT seems to be more holistic than linear. This explains why, even if a specific skill or knowledge is prioritized or worked on deeply, there will always be a CT expert from whom good reasons could be argued to indicate that a wrong path has been chosen.

CT, as a holistic discourse, seems to have great potential in the field of education. For this reason, severe criticisms arise, demanding that CT apply to itself the criteria it strongly defends (Mulnix 2012). These criticisms seek to commit CT not only to cognitive changes in the student but also demand that it be operational, that is, that it can offer useful tools to effectively solve real-world problems (Atanasiu 2021; Halpern and Dunn 2021; McLaren 1994).

How can we design an intervention program that allows students to develop CT skills? How can we ensure that these skills are not only applied in the academic field but also generalized to the real world? How can we make CT a useful tool for solving everyday problems, both personal and professional? Despite the importance given to CT in pedagogical discourse, these questions highlight existing gaps. The lack of effective answers is the reason why CT is sometimes perceived as a grandiloquent discourse that is disconnected from real-world problems. Perhaps this has not been intentional but simply the result of prioritizing some attributes over others. The truth is that this approach, characterized by argumentation theory, has been criticized by experts who consider that CT fails to connect thought with action (Barnett 1997; McLaren 1994; Walters 1992). It seems that the term ‘critical’ remains in the abstract separation of assumptions and facts, but the latter loses prominence. Alternatively, it is as if the programs focused only on cognitive objectives, making the student aware of something important and necessary to know and handle but not necessarily to apply.

For this reason, authors like Barnett (1997) chose to refer not to CT but to critique. According to Barnett, critique is structured in three dimensions. The first is linked to the analytical stage of evaluating phrases or arguments. The second is associated with an act of a metacognitive nature: self-regulation and self-examination. Finally, critique action is the realization of the will to analyze and evaluate the real world. This is aligned with an ideal of transformation that can be individual or collective, such as an ideal of social justice that can make this a better place. Therefore, critique, understood as the conjunction of the self and action, allows us to speak of a fully critical person. This makes a lot of sense to develop a CT that transforms reality and provides tools to solve problems in the real world. Paul (2018) has also questioned this traditional approach and has proposed distinguishing between weak or narrow CT and strong or broad CT. The weak CT defends, analyzes, and evaluates a set of propositions or arguments within a limited framework. Arguments that belong to that framework are considered solid or valid, and dissenting opinions are presented as fallacies. On the contrary, strong CT conceives the thinker as someone capable of transcending their framework, accepting that there can be more than one valid argument, even if they do not agree with it. This implies that CT is not only associated with the quality of judgment on the argumentative or exclusive reflection with the linguistic components but also with the referent, the real world.

In this line of argument, a CT approach that places the real world as the protagonist faces a complex task. First, it must be based on a CT concept that recognizes and connects the logical–epistemological, cognitive psychological, and critical pedagogical traditions. It is important to recognize that the conceptual heritage has had representatives from each of these branches, and the CT cannot be simplified to a single constitutive definition. The goal is to find an operational concept that can measure the skills subordinate to the student’s operational change, allowing him to solve real-world problems through generalization.

Secondly, the program that is built on this concept must also recognize and methodologically resolve its limits. In this way, the CT can move to dimensions that are different from the logical–epistemological, where argumentation has prevailed as a keyword (Jahn 2019; Jahn and Kenner 2018; Saiz 2017, 2020). Without detracting from other programs that have focused all their efforts on theories of argumentation (Morrow and Weston 2016; van Gelder 2015; Walton 2000) with exceptional achievements, making it one of the skills with the most consensus in evaluative criteria, the concept of CT we are looking for must have a different gravitational axis. As can be seen, the change in focus is not minor, and the proposal is not limited to simple descriptions. It is about highlighting the interests, personal and professional problems, as well as the social needs inherent to the individual under the conception of the CT as a theory of action. The idea would be to place social life (the facts, the behaviors, etc.) as a unit of analysis and questioning at the center of a CT instructional program to discover tensions and inconsistencies between perspectives that make the CT more original or closer to an individual with economic, cultural, political, but also everyday and professional interests.

How can we advance in a challenge that we know cannot be met with an intervention program due to the inherent complexity of (CT)? Despite the obvious limits, how can we implement an instructional program that focuses on the demands of the real world without losing methodological rigor and the historical richness of CT? Finally, how can we determine if our conceptual, methodological, and generally instructional efforts are effective in addressing the realities of the world?

This article presents the ARDESOS-DIAPROVE program, which seeks to respond to the demand for a change in focus in (CT). The program does not intend to be a definitive solution but a pedagogical effort to develop, strengthen, and promote CT in relation to the real world. Our thesis argues that CT should focus on the best explanation and not necessarily the best argumentation. Given its trajectory, background, and consolidated quantitative results, it can be considered that ARDESOS-DIAPROVE is a mature program that has been evaluated in different scenarios and is constantly improving (Rivas and Saiz 2023; Saiz 2017, 2020; Saiz and Rivas 2011).

We will focus on one of the key foundations of the program: explanation. From an epistemological perspective, we seek to place explanation at the center of the program as a possible response to a shift in focus in CT. Given that explanation has an inherent causal nature that interrelates with the real world, it is a key concept in the objectives of science. In the program, the importance of explanation strategically displaces argumentation. The methodology of CT focuses on finding the best explanation for professional and everyday problems to make accurate decisions. Logical support and the causal explanatory model are used to privilege uniqueness. These are based on the inference to the best explanation (IBE) (Galavotti and Pagnini 2010; Harman 1965; Hitchcock 1995; Lipton 2003; Salmon 1984a, 1984b) and on the inference to the only explanation (IOE) (Bird 2007, 2010).

Identifying and studying each of the foundations of the program, including those of the epistemological spiral, is part of the program’s continuous improvement strategy. This includes increasing clarity, providing didactic examples, updating concepts, and debating the main challenges proposed by epistemology. In addition, the program incorporates epistemology, a central branch of philosophy that can significantly contribute to scientific literacy, a trait that should also be part of the critical thinker. After all, the epistemological debate focuses, among other things, on the true and justified beliefs we have about the real world (Bunge 2000).

## 2. ARDESOS-DIAPROVE Program

CT is one of the higher thought processes (Paul and Elder 2006). Its complexity lies in the fact that it relates cognitive, attitudinal, and metacognitive components (Lau 2015; Rivas et al. 2022). Conceptual comprehensiveness and its implementation lead us not only to focus thematically on the development, strengthening, and promotion of some skills, but we must also deal with those other logically necessary components that function as the engine that mobilizes skills towards results. Thus, for example, if, as in our case, we privilege specific skills intrinsic to reasoning, decision making, and problem solving, we must also privilege non-cognitive components, such as the will to do it, really desire to put into practice the necessary and useful problem-oriented reasoning. This conceptual network is more evident in operational conceptions of CT, such as those that focus their efforts on establishing an identity between the critical and the effective, since willingness to be effective is determined by intrinsic and exogenous motivations so dissimilar and multiple that they are complex to systematize in the conceptual network of a definition. Thus, metacognition, self-regulation of skills, and motivation, the active driving force behind them, form an essential part of the program (Saiz 2020).

We think to solve problems, we make our inventive and problem-solving efforts available in the face of epistemic, ontological, ethical, and political obstacles or concerns (in general, in the face of a problem). This means that we plan, strategize, and act to achieve that solution. Decision making is key in linking the start and end of any process. It is not just about finding the best theoretical solution but also about ensuring it can be applied in reality. This creates a complex network of concepts that seem separate only in theory.

As a corollary to this conceptual framework, we have chosen to characterize critical thinking, rather than as an academic discourse, as a tool for effectively solving a problem or achieving desired goals; moreover, as a generator of change, understood as the passage from one welfare state to another. This establishes serious commitments, as it means that no effort must be spared in achieving the effectiveness of the means (the best result, the best explanation, or problem solving) to accomplish the desired change. It also implies that it is not enough to find alternative answers or solutions but to find the best among the range of options. If thinking critically is thinking effectively to achieve the desired change or goal, and this is not only desirable in the academic and professional sphere but extends to the everyday and personal sphere, CT is desirable higher order thinking. Therefore, by virtue of the conceptual network described and the relevance of critical thinking, we have proposed that “to think critically is to reach the best explanation for a fact, phenomenon or problem in order to know how to solve it effectively” (Saiz 2017, p. 19).

### 2.1. Methodological Foundations

The program has been modified and is the product of several years of research from its origin to the present, giving positive results and continuous improvement, as can be seen in different works (Rivas and Saiz 2023; Saiz 2020; Saiz and Rivas 2011). ARDESOS can be understood as the program’s mesocurriculum, as it includes everyday situations to accommodate the activities of problem-based learning (PBL) or ecological learning (Morales et al. 2015), the argumentative tradition, decision making, and problem solving. At the methodological and didactic level, the foundations are the development of DIAPROVE. Firstly, it consists of identifying the limits of a good thinker, i.e., identifying the deficiencies and biases that prevent progress in the development of critical thinking. This is key, as we are faced with errors that can be systematic and prevent us from thinking correctly, as well as ontological and epistemic compromises that can block the search for the best explanation, such as irrelevant or isolated but striking data that can make us lose sight of the objective and create a blanket of contradictory data or details that need to be observed and analyzed (Saiz 2020). This part is therefore crucial, as it can prevent correct diagnoses from being made. After this, the search for the best explanation that can explain the facts and forecast is ensured by the suitable application of logical principles, causal simulations, and other necessary contrastive steps.

In short, the general route of the program is to identify the cognitive biases and deficiencies that sometimes prevent us from solving some problems, looking for the best explanation of the problem, and making sure it is the best. The following are some of the most relevant foundations of the program:

(a) We think in order to survive. Biological nature determines what we are on a physical and mental level, the latter feature emphasizing that the essential mental actions (perceive–learn–retain) are a function of the *telos* as a species: to survive (Saiz 2020).
(b) Thinking, willing, and acting are inherent actions. If (a) is accepted, then the concept of “thinking” does not refer to just any word but to critical and effective thinking: it is not about finding the solution but the best solution. However, the links between thinking and acting are the non-cognitive components of CT (dispositions, motivations); therefore, neither skills nor dispositions alone are sufficient for a CT program (Saiz 2017).
(c) Thinking is inferential. The best solution can only be arrived at by a correct inferential process, which, within its own complexity, contains instances of contrast (verification/falsification) with the facts.
(d) The deductive machinery is necessary, though not sufficient. It is necessary because it warns us and helps us to correct our biases and cognitive limitations. Understanding the complexity of the inferential process (c) requires knowing the basic rules of logic and adopting a more semantic than syntactic stance so as not to confuse the sufficient condition with the necessary condition and vice versa. However, it is neither necessary nor sufficient, as the formal scaffolding must be complemented by causal scenarios to be resolute (Saiz 2020).
(e) The connection between CT and metacognition is reciprocal. Critical thinking does not arise randomly; it only manifests when we are aware of our actions, whether they are correct, to repeat and improve them, or incorrect, to amend them (Rivas et al. 2022).
(f) It follows that metacognition is what enables us to be aware of our cognitive process and, in turn, to transfer it to other areas of life, to reflect on how we learn, and to select, monitor, and evaluate our own strategies.
(g) From (e) and (f), it follows that the program incorporates the non-cognitive component (b) through the treatment of metacognition and motivation, which are sequentially linked to each other since skills can only be organized, planned, and goal-directed once they have started to function, i.e., motivation must activate skills for metacognition to function.
(h) For this last reason, and to bridge the gap between theory and practice, sometimes criticized in intervention programs, or what in practical terms is the gap between training and what the professional environment demands of students (Casner-Lotto 2006), the program uses relevant situations so that students have the possibility of applying what they have learned (Saiz and Rivas 2008). Under the premise that we can only know what we apply and that there is no point in perfecting critical skills if they are not used, the activities characterized as relevant in the program are based on PBL (problem-based learning). This is mainly because it is problem-based learning in which the student must apply processes of research, reflection, and analysis that are evident in the delimitation and understanding of the problem, interpretation, decision, and, obviously, solution.

### 2.2. Epistemological Foundation

The first distinctive feature of the program is that it distances itself from the logical–epistemological tradition if the latter is reduced solely to the theory of argumentation. What we have argued in this respect is that CT is at the service of the best explanation and not of the best argumentation as an end in itself. This break is better understood if we consider the dilemma of understanding CT as a theory of argumentation or as a theory of action (Saiz 2017, 2020).

As a theory of argumentation (Hitchcock 2017; Morrow and Weston 2016; Toulmin 2003; van Gelder 2015), the identity between knowing or training in CT and achieving goals inherent in human life itself (such as happiness or well-being) is questionable. As a theory of action, the identity between knowing and solving effectively in practice must be a principle. This nexus is unquestionable in a theory of action; it is not enough to make the argument the best product of reasoning, but at most, a feasible means to ends that require problem solving.

The aim of the program is to achieve effectiveness through the best explanation. In the face of a fact, explanation takes precedence over argumentation. Through the description of the process and the incorporation of strategies of order and clarity, the latter appears to justify what is found in the explanatory process; it proceeds to discursively substantiate why it is that cause and not another. Thus, argumentation is subordinated to explanation. The conceptual and operational linkage of the critical concept is with the indicator of effectiveness; one is effective to the extent that one is talking about a better way of doing things, which means that it is not possible to be critical and not effective.

The focus of the program is on explanation, which means that it is a core concept around which the other concepts of the method orbit with multiple relationships to achieve change as an objective of critical thinking. Explanation is the heart of the program due to its direct link with reality. Imagine a situation where there is compelling evidence but no response to a phenomenon that is not explained by our current beliefs. To understand and solve this, we need to identify the cause.

Argumentation requires activating this response. But even so, outside the problem, argumentation could appear in a simulated situation or to sustain a conviction or belief that does not arise from a problem of reality. Explanation as a concept proper to inquiry is conceived as a mechanism that engages with the deductive machinery to establish the combinations of deductions with the facts and to unconfirm alternative explanations so that the objective of CT is fulfilled, namely, to find the best explanation and with it, once the decision making takes place, to bring the explanation as a solution into the practical realm (Saiz 2017, 2020).

A program like ARDESOS-DIAPROVE can only be nourished by science, its data, and methodologies. Science is distinguished by the constant search to understand and explain the phenomena we observe in the world. Studies of scientific practice show that it is not limited to pure observation but often involves the search for testable hypotheses. This activity is carried out with the precision of data obtained through experimentation (Bunge 2000). For this reason, science describes events and links seemingly unconnected propositions into a coherent system of explanations. This ability to link and abstractly formulate structural properties is what differentiates science from common sense and elevates it to a deeper and more systematic level of understanding (Nagel 1987). With the conviction that it is not simply a matter of observing and recording but of understanding, of looking for patterns, of formulating hypotheses, and testing them, science advances by providing explanations that are both rigorous and capable of refinement (González and Guamanga 2022). This commitment to explanation defines and distinguishes science and is the reason it determines the program.

Scientific explanation is a crucial tool in the development of critical thinking. By understanding how foundations in science are constructed and validated, it is possible to strengthen essential skills for logical reasoning and the evaluation of claims. In this section, we will focus on three epistemological approaches to scientific explanation: the theories proposed by Wesley Salmon, the concept of IBE, and IOE. Through this analysis, we intend to provide some of the epistemological foundations of the ARDESOS-DIAPROVE program, especially those dealing with the scope and limits of explanation. This is to deepen the challenges to be considered when looking for ways to develop the ability to explain to promote critical thinking. For this, it is significant to distinguish between the conceptualization of explanation and how it is achieved and, of course, to establish that a request for an explanation of an everyday or professional situation is not necessarily a scientific explanation but an explanation that emerges from the epistemological standards of science.

#### 2.2.1. The Spiral of Explanation

Wesley Salmon has been a leading figure in the debate on scientific explanation (Galavotti and Pagnini 2010). His approach focuses on the causal nature of explanation, a perspective that seeks to go beyond classical analyses, as those proposed by David Hume (1739). In the *Treatise on Human Nature*, Hume puts forward an empiricist view of knowledge, arguing that many fundamental ideas, such as causality, have no direct impression to derive from and are therefore mere chimeras. With the example of billiard balls, Hume questions the traditional notion of causality, arguing that we cannot perceive a necessary connection between events but only certain circumstances: contiguity, priority, and constant conjunction.

Contiguity refers to the specific order in which we perceive events in space and time. Priority indicates that cause always precedes effect, although this does not imply a direct causal relationship. Constant conjunction suggests that, given certain conditions, the same effects will follow the same causes. However, Hume argues that, beyond these circumstances, we cannot discover a necessary connection between cause and effect. In his view, our idea of causality arises from habit and the expectation that the future will resemble the past rather than from necessary connections.

Whereas Hume regards causality as an epistemological and psychological construct based on habit, Salmon (1984a) tries to establish the existence of objective causal relations in the real world. Salmon’s aim is to show that these causal relations can satisfy the explanatory demands of science. For Salmon, scientific explanation goes beyond the simple connection of relationships, so he proposes that a true explanation must identify complete causal processes and structures. This perspective differs from other approaches by stressing the importance of objective causality rather than simply observed regularities.

Salmon stresses that not all causal relations are compatible. For a causal relation to serve as an explanation, it must be part of a complete causal structure. This structure must be able to show how particular events or conditions give rise to the phenomena being explained. Salmon proposes an alternative notion of causality that aims to satisfy the demands of explanation in science. Unlike Hume (1739) and Mackie (1965), Salmon suggests that it is not adequate to speak of “the cause” of an event since an effect can have multiple causes. Instead of presenting individual causes, Salmon introduces the concept of “causal process”, which refers to the transmission of an effective entity, such as energy, information, or electric charge (Salmon 1984a; Gómez and Guerrero 2020).

Salmon uses Newton’s theories to reconstruct Hume’s illustration of billiard balls, contending that physical laws offer an objective method to evaluate the relationships between objects. He presents the idea of causal interaction, a term used to describe scenarios where processes of cause and effect intertwine, leading to enduring transformations. He further underscores the phenomenon of mutual modification, which transpires when two such processes intersect, resulting in reciprocal changes. An illustrative example is the collision of two billiard balls: each trajectory is a causal process, and the collision is a causal interaction. If a ball bears a mark, such as a scratch, and continues its trajectory after the collision, it demonstrates the continued transmission of the mark, reaffirming the causal nature of the process (Salmon 1984a).

Finally, Salmon’s causal mechanistic model of explanation is based on several key points. These include the distinction between causal processes and pseudo-processes, the counterfactual notion of transmission of a mark, and causal interaction. Explanation within this model traces the causal processes and interactions that lead to a specific event, allowing for a deeper and more nuanced understanding of causality in various phenomena. In this model, a causal process is a physical phenomenon that can transmit a “mark” on a continuous basis. These processes are distinguished from pseudo-processes, which cannot transmit marks. In addition, Salmon introduces the idea of causal interaction to account for two causal processes that intersect and modify each other. In the model, explaining an event involves tracing the causal processes and interactions that lead to it (Gómez and Guerrero 2020). Although the model of explanation proposed by Salmon succeeds in accounting for many phenomena and, in some respects, overcomes the problem of causality proposed by Hume, it is not without its critics. In this regard, Christopher Hitchcock (1995) argues that Salmon’s model does not fully capture the essence of what we consider relevant in an explanation. For example, in the case of billiards, although the model may identify the movement of the balls as a causal process, it does not distinguish which aspects of that process are, in fact, relevant to the explanation, such as the mass and speed of the balls, as opposed to other less relevant factors.

Christopher Hitchcock (1995) also points out that the model faces similar challenges in other contexts, such as the example of birth control pills. Although taking a pill can be considered a causal process, the model does not adequately distinguish between the relevance of taking a pill for a man (which is irrelevant to preventing pregnancy) and for a woman (obvious relevance). Thus, Salmon’s model, while useful for identifying causal processes, does not provide an adequate tool for discerning which features of a process are actually relevant to an explanation.

Another significant criticism focuses on the inclusion of pragmatic and contextual elements in his model. Although Salmon attempts to establish a notion of objective causality, his proposal seems to compromise this objectivity by relying on the interests or views of users. To illustrate this point, consider the example of the 40-year-old man who faces a series of unfortunate circumstances on a stormy morning. His old car, the storm, the bad road, the faulty brakes, his work-related stress, and the lightning striking a tree all contribute to a tragic accident. Hours later, three experts arrive on the scene: a medical examiner, an insurance mechanic, and a road engineer. Each, from their professional perspective, could identify a different cause of the accident. The doctor might point to a heart attack, the mechanic might focus on faulty brakes, and the engineer might criticize the road design. In this case, the objectivity of causation is challenged by the different perspectives and areas of expertise.

The ARDESOS-DIAPOVE program tries to take the best of existing theories of explanation and adapt them to meet the specific needs of its pedagogical approach. In this case, the program emphasizes, from Salmon’s approach, the importance of identifying objective causal relationships in the real world. The distinction between causal processes and pseudo-processes provides a valuable tool for the program, as it allows for a deeper understanding of facts beyond appearances. However, the program cannot follow Salmon’s model point by point, especially as it opens the door to different perspectives, leading to different explanations. Moreover, although the mechanistic causal model of explanation is useful for identifying causal processes, it does not always provide an adequate tool for discerning which features of a process are relevant to an explanation.

Salmon’s model focuses on causes and stipulates necessary features of the explanation, such as showing which factors are relevant for the occurrence of the event and excluding irrelevant factors; however, the central difference with the program is that in Salmon’s model, the event is placed in a network of statistical relationships of factors that are relevant for its occurrence without this implying finding the best explanation. While Salmon’s model does not conceive the explanation under the structure of an argument and the fact remains in a geometric network of relevant interactions, from the ARDESOS-DIAPROVE program, it can be questioned, in Galavotti’s words (Galavotti 2018), that it is still not indicated which properties should be taken as explanatory, it is like a telephone network that does not account for the specificity of the messages, while in the program it is crucial to get to the message, to the best explanation.

#### 2.2.2. The Inference to the Best Explanation: Better?

IBE, first proposed by Gilbert Harman in 1965 and later extended by Lipton (2003), emerges as an essential epistemological methodology in hypothesis selection in science. Harman argued that not all inductions are based on guarantees of non-deductive inference. Instead, it is necessary in many cases for scientists to infer a hypothesis not because it is possible but because it provides the best explanation for the available evidence (Harman 1965). Unlike traditional approaches that rely exclusively on direct observations or deductive logic, IBE foregrounds the explanatory power of a hypothesis.

This intrinsic process of IBE begins with the identification of hypotheses that might make sense of certain evidence. Given the plurality of hypotheses that can often explain the same evidence, it becomes imperative to evaluate and test these hypotheses against each other. Harman emphasized that the choice of the “best” hypothesis is not an arbitrary act but is governed by specific criteria. These criteria include aspects such as the simplicity of the hypothesis, its ability to address and explain a variety of phenomena, and its consistency with the established body of knowledge.

To further illustrate how this process works in practice, consider the following example: in a small village, the eucalyptus trees, symbols of the locality, started to become diseased, with dark spots on their leaves and discoloration of their bark. The scientists proposed several hypotheses: H1, a new pesticide affected the trees; H2, an unknown fungus infected the eucalyptus trees; H3, climatic changes stressed the trees; and H4, invasive insects damaged the eucalyptus trees. After evaluation, only hypothesis H2 was retained, supported by the presence of a fungus in diseased trees that was absent in healthy trees.

To validate hypothesis H2, the fungus was introduced into a healthy tree in a controlled experiment, and the tree began to show symptoms similar to those of the diseased eucalyptus, confirming the cause of the disease. This process demonstrates the effectiveness of IBE in scientific research, highlighting the importance of rigorously proposing, evaluating, and validating hypotheses in order to reach accurate conclusions (Laudan 2007).

The IBE has established itself as a valuable tool in the scientific landscape by enabling researchers to deduce the most likely cause behind an observed phenomenon. This methodology is characterized by a series of criteria that determine what is considered a “best” explanation. These criteria, which include simplicity, consistency with established theories, explanatory scope, and plausibility, allow scientists to select hypotheses not only on the basis of observations or pure logic but also on their intrinsic ability to provide a coherent and plausible explanation in the face of the evidence presented. IBE is an essential methodology in science that guides researchers in selecting hypotheses based on the available evidence.

However, this tool is not without its critics. One of the most prominent objections is the “bad lot” argument, which postulates that all available hypotheses may be inadequate in certain circumstances (Fraassen 1989). An illustrative example, following the eucalyptus case, where a hypothesis not initially considered turned out to be the true cause of a disease (H5: a specific type of water contamination affecting eucalyptus trees), shows that the original hypotheses were insufficient.

IBE faces epistemological challenges, especially when it comes to inferring truth from criteria such as simplicity and coherence. While these virtues are useful for assessing the quality of an explanation, they do not guarantee its truthfulness. Therefore, while IBE remains central to scientific reasoning, it is crucial to recognize and address its limitations and potential with a critical approach.

The ARDESOS-DIAPROVE program, like IBE, values the importance of explanation as a central tool for understanding and solving problems. Both seek to identify and validate the best possible explanation for a given phenomenon or problem. However, while the IBE focuses on virtues such as simplicity and coherence to judge the quality of an explanation, the ARDESOS-DIAPROVE program emphasizes the practical and applied relevance of explanations and, above all, the process of eliminating possible explanations to obtain not only the best but the only one that can explain the event.

#### 2.2.3. Inference to the Only Explanation: The Only One?

The difficulties that conceptually arise have tried to be studied by advocates of this inferential process characteristic, according to them, of science. Among them is the philosopher of science, Alexander Bird (Bird 2007, 2010). Bird’s proposal moves from IBE to IOE. For our purpose, understanding this approach is key, as the correlation with the ARDESOS-DIAPROVE program would be in the central thesis, unlike the relationship with Salmon and the IBE, with which relationships are established between some premises.

Bird’s thesis is that if the IBE allows the selection of a single potential explanation, then that is the only explanation (Bird 2010). He is, therefore, committed to the uniqueness of explanations. Bird (2007) calls this inference “Holmesian inference”.

By inference to the only explanation (IOE), I intend something quite specific: at the end of the inquiry, we can be in the position to infer the truth of some hypothesis since it is the only possible hypothesis left unrefuted by the evidence. It is the form of inference advocated by Sherlock Holmes in his famous dictum, “Eliminate the impossible, and whatever remains, however improbable, must be the truth”. Of course, one requires the auxiliary hypothesis that there is an explanation of the phenomenon in question (p. 425).

It is an IOE because all but one explanation can be eliminated from the evidence. In structural terms, we would have an argument that goes from determinism, through hypothesis selection and eliminative inference, and ends in a single conclusion. In this way, it seems a more categorical bet than the Hartman–Lipton IBE by not resorting to other explanatory goodness, and therefore, Bird will argue this initiative through scientific practice in medicine, for which he reconstructs the classic case of Semmelweis.

Bird’s interpretation does not supplant Lipton’s explanation (Lipton 2003) but shows the complement of the IBE in Semmelweis’ case. For Bird, this case obeys more a Holmesian inference than a Liptonian IBE, and as proof of this, the “explanatory charm” of the inferential study can be omitted. Holmesian inference structurally starts from a fact that has an explanation; there is a collection of hypotheses that can explain it, and the hypotheses have been falsified by the evidence, except for one. It follows that the hypothesis that survived the eliminative process is the hypothesis that explains the fact. As can be seen in the scheme, there is no other criterion or explanatory goodness beyond having passed the process of testing against the evidence.

In following Lipton’s approach, Bird also accepts the basic IBE principle of discarding the null or zero hypothesis, i.e., the deterministic idea that for every intriguing piece of evidence, there is an explanation. Bird emphasizes the process of progressive refutation, i.e., eliminating rival hypotheses until the true explanation remains, hence the name Holmesian inference: “Eliminate the impossible and what remains, however improbable, must be true”.

Bird’s case study is Semmelweis. Between 1844 and 1848, Semmelweis worked at the Vienna General Hospital. The scientific concern that made him famous and a reference in epistemological studies was why a large number of women contracted a terrible and sometimes fatal disease called puerperal or postpartum fever with a higher preponderance in one division than in the other. The investigation led Semmelweis to consider various hypotheses, from the most scientific to the least plausible, each of which was tested by experience.

The difference with other analyses is traced by Bird in determining what is selected in this case as intriguing evidence (explanandum) to understand the correspondence of this with the *explanans*. Either E1, “The existence of puerperal fever in division 1”, or E2, “The preponderance of puerperal fever in division 1”. The former is much more general, as it can be understood as speaking of the existence of the disease elsewhere. The following hypotheses emerge from the first phase of the IBE: H1, the cause of the high mortality is the overcrowding of division 1; H2, the cause is an epidemic or climatic influence; H3, the culprit is the careless examination of medical students in division 1; H4, the cause is the terrifying effect of the priest passing through division 1 before anointing dying patients; and H5, the cause of the disease in the division is that women gave birth on their backs. Once the possible *explanandum* and *explanans* have been determined, it is essential, Bird points out, to determine the *explanandum* to correctly understand the phases of the IBE. If, for example, it is E2, H1 and H2 can be ruled out since divisions 1 and 2 share much of the same healthcare practice conditions and, division 1 being infamous, then division 2 was more overcrowded. In the contrastive explanation, there is no particularity that is unique to division 1. According to Bird, H3 is an implausible hypothesis that even Semmelweis could dismiss based on initial knowledge, namely that there was no significant difference between the care of students and midwives in division 2 versus natural childbirth injuries. In the case of H4, Semmelweis convinced the priest not to go through division 1, and this was not significant with the results: the large preponderance of postpartum fever, i.e., the evidence was inconsistent, and H4 can be falsified. The same was true for H5, given that women gave birth sideways without this constituting a decrease in mortality. This means that no H can explain E2. The case that accidentally made another hypothesis possible was the death of a colleague of Semmelweis, Dr Kolletschka, who died of a wound sustained during an autopsy. Semmelweis observed that Kolletschka’s symptoms were similar to those of women dying of postpartum fever. This allowed him to posit H6: the women were infected with “cadaveric matter” by medical students performing autopsies before examining the women in division 1.

In this regard, Bird states that, from this hypothesis, there are two divergent hypotheses, only one of which is supported by the evidence and the Kolletschka case: the first (H6a), the women were infected during the examination by the medical students; and the second (H6b), the infectious agent was the “cadaveric matter” imported by the students. Bird’s claim is that only H6a is verified by the evidence, while H6b is plausible, but with the data, it cannot be verified; otherwise (bearing in mind that Semmelweis suggested that the students wash with a chlorinated lime solution and this measure caused the deaths to decrease), the cause lies in some property of the students’ hands that is removed by washing them. With this, Bird asserts that the evidence leads not only to the best explanation but sometimes to the only explanation and that it is arrived at by eliminating all potential alternatives. From this statement, it is relevant to note, “sometimes”, to limit the claim of universality or extension to all phenomena in science.

The ARDESOS-DIAPROVE program focuses on finding the best explanation for professional and everyday problems involving decision making. Moreover, it has a logical underpinning and a causal explanatory model. Thus, the inference referred to is not in an epistemological gap but is an inference that seeks uniqueness in the same way that Bird has proposed. Now that we have an identified foundation with which there are conceptual and methodological relationships, what does the program gain by identifying an epistemological foundation? From the perspective that this is a program underpinned by scientific data and validated pedagogical practices, the gains can be debated. What is unquestionable is that it provides greater conceptual clarity and foundations to a program whose didactic route is to achieve better results through Holmesian inference, as we have pointed out on other occasions, at that point without the need to descend theoretically down the explanatory spiral. On the other hand, the search for the causes in the science of phenomena or problems in which life is at stake is a component that enriches the program didactically. Within the didactics, a unit on these achievements in science and how they have been a successful illustration case shows the strength of how we are in line with the most accurate description of science and how a professional also solves problems effectively.

The points of agreement with Bird that can be noted are as follows: first, the premise that every phenomenon requires an explanation, and this explanation is causal, material, and objective, is not disputed. Bird’s analysis of the classic Semmelweis case illustrates this: faced with a multiplicity of hypotheses, some of them dominated by a set of unfounded beliefs, they are dismissed by the inscrutable strength of the facts. Secondly, both in Bird and in the program, there is this need to arrive at the best explanation through the procedurally correct elimination of logical principles in combination with the contrast with experience. In Bird, we can also observe the need, as in our program, to attend to relevant, contradictory, and extraneous data in order to propose an alternative solution, a reason that resembles the ongoing process of knowing how to handle data with the deductive machinery. Thirdly, although Bird accepts that his case of arriving at a single explanation is an exceptional case of the IBE, this is not a limit to the program; as Bird detailed above, the issue is not understanding the problem but determining which *explanandum* is the one to be solved. It may be the case that curricular activities do not have this causal relationship or that they are, in principle, posed as multi-causal when, in fact, the question or problem has not been adequately defined.

Finally, it can be seen in Bird’s proposal that his cases are set in reality and not in the imaginary; he shows cases that happened and were transformative for some sciences. In the same way, the program proposes real activities of ecological situations that, if not resolved, have implications in the real world. It can therefore be concluded that we are faced with an epistemological reference that also supports the program’s proposal. This is certainly not costless, as it requires a deepening of the reserves as the bad lot to avoid in the hypothesis construction phase to ensure that you really understand the problem to be solved. There is, therefore, more common ground between Bird and the ARDESOS-DIAPROVE program than with other proposals. The limits pointed out are precisely pedagogical warnings so that the didactic material and instructions, the PBL, and other pedagogical foundations are well-aligned curricularly with the purpose of the program.

#### 2.2.4. Conceptual Circuit

In the ARDESOS-DIAPROVE program, the conceptual circuit that complements the IOE is based on heuristics and decision making. An epistemological pillar may have little value if it fails to apply to real-world problems, becoming a mere static ornament. It must establish cooperation networks with other concepts to achieve an effective connection with reality where decision making stands out as the key concept.

The definition of CT that we use as a starting point for this program has a clear intention: to find effective solutions to problems. However, it is evident that finding solutions and executing them are two different things. The IOE, along with its entire process, provides us with a guide on how to reach these alternatives and the criteria to choose one of them. This is a crucial step, as it ensures rigor and transversality in the search for an explanation. Ideally, the process should go beyond the abstract solution of the problem, and transversality should be evident in a well-made decision. However, the principle of reality about how we make decisions, especially when we collect data about our mistakes when deciding, confronts this ideality. The issue of the limits of our cognitive machinery arises in the program with the same naturalness with which we accept our fallibility. For this reason, we have decided to integrate real cases into the conceptual configuration. These cases are designed to challenge the student to face future professional problems, some of which will require transcendental decision making in practical terms. These decisions will not only test the professional quality of the student but will also influence the decisions that others will make based on the professional’s criteria. To illustrate this, we will use the Lilly case (Saiz 2020).

Decision making is a skill that is perfected after having made the decision. There is a path, whether intuitive or rational, that has been followed to select and carry out a solution. It is possible that a specific alternative has been chosen as a result of the extensive path described in the IBE process or that this path has been shortened. In any case, the decision arises as a result of the application of one or several pre-decisional skills. To a large extent, the dilemma determines the quality of the decision: the greater the identification and monitoring of pre-decisional skills, the greater the self-regulation in case it is necessary to intervene to correct any error in decision making.

The high recurrence of failures in decision making could contradict the essentialist definition of man as a being given with logos: language and reason (Gadamer 2004). However, this definition is quite far from the results proposed by Kahneman and Tversky, who, through extensive experimental work, demonstrated that there is no such rational purity or essentialist category of rationality (Kahneman 2013; Kahneman et al. 2022; Kahneman and Tversky 1984; Tversky and Kahneman 1974).

According to the data, the opposite occurs: in different professional scenarios, where agreements and correct decisions should predominate, such as medicine and law, decisions are easily classified into random scatter plots (noise) or systematic deviation (biases) (Kahneman et al. 2022).

What is the role of CT in the face of obstacles that lead to wrong decisions? The answer seems clear: it must incorporate them into an instructional design to expose students to these deficiencies of cognitive machinery, both at a descriptive and normative level. This exposure should facilitate the identification of these deficiencies and the generation of strategies to reduce them in the decision-making process. The program takes on this challenge and, through specific cases, exposes the student to situations in which, if they do not overcome these deficiencies, they will not be able to solve the problem and, as a result, will probably make a wrong decision. In this thematic content, the explanation again takes precedence over the argument. Although the latter could arise even after reaching a wrong alternative, the explanation directly precedes decision making. The action of arguing can be activated even in defense of a wrong decision. Given a point of view that wants to be defended and an intention, it is possible to develop an argument with a logical emphasis, oriented to the quality of the relationship of the propositions, dialectical, focused on the correct dialogic processes, or rhetorical, focused on the universes of beliefs accepted between the speaker and the audience (Tindale 2004). However, these approaches do not correct the possible error but can amplify it. Therefore, there must be other paths that lead us to decision making or on which the hygiene of decisions falls (Kahneman et al. 2022).

Hygienic decision making is based on disciplining intuition. It is not about prohibiting it but resisting premature intuitions and balancing them according to the context that requires explanation, factual data, causal scenarios, and forecasts. These are necessary steps, among others, to arrive at the only explanation (Saiz 2020). It is important to distinguish between the generation of explanation alternatives and the choice between them. Although the distinction is simple, clarification is necessary to avoid confusing problem solving with decision making (Halpern and Dunn 2023). The central point is to have and master clear and solid criteria for choosing an alternative and, in a strict sense, excluding the others, thus advancing towards decision making with an explanation alternative as the protagonist. These criteria and procedures have been provided to us by the epistemological debate in this article illustratively by IOE.

From an analytical point of view, it could be argued that without the best and only explanation, we are unlikely to be making a good decision. This statement supports the inclusion of heuristics and biases in the program, in particular, the representativeness heuristic. According to Kahneman (2013), “The technical definition of heuristic is a simple procedure that helps find adequate, though often imperfect, answers to difficult questions” (p. 271).

This suggests that heuristics can lead us to make incorrect judgments and establish a hierarchy of events that is not necessarily based on solid foundations. An example of this is the representativeness heuristic, which uses a personality trait as a basis to estimate the probability of expected behavior, giving priority to this trait as if it were representative of the entire personality structure (Saiz 2020).

With the aim of completing the conceptual cycle of the ARDESOS-DIAPROVE program in relation to IBE, real-world decision making, and the representativeness heuristic, we propose the Lilly case for integrated analysis. In this case, the tragic death of a company manager occurs under suspicious circumstances. The manager’s sister, also a co-owner of the company, plays a crucial role. With a history of mental health problems (depression, suicide attempts, emotional independence) and the consumption of sedatives on a tragic night, she shoots her brother, mistakenly believing he was an intruder in the house they shared. Despite her acquittal due to the accidental nature of the incident, questions arise about her ability to lead the company and her possible involvement in the fact. As the new manager, she has made significant business decisions without consulting the board of directors. The same happens in her personal life; she has set the date of her wedding without consulting her fiancé. The company’s executive board commissions a psychology professional to prepare a report to determine whether or not the new manager is qualified to run the company. The professional is asked to decide with an exclusive response, yes or no.

The professional is at a crossroads to make a decision. Let us analyze the first path, which originates from the representativeness heuristic. By focusing on personality traits such as emotional dependence and low self-esteem during the patient’s study, it is natural to conclude that she is a person with a tendency to self-harm. Evidence of a suicide attempt could reinforce this assessment, even leading to rule out that she is prone to harm others. By establishing a personality profile based on the observed traits and behaviors, the professional could overlook relevant information, such as the patient’s involvement in the accidental or intentional death of her brother. As a result of this diagnosis, the professional would consider it unlikely that she is an aggressive and violent person, as the observed traits are representative of certain behaviors. The professional’s categorization can cause other relevant data to go unnoticed, focusing only on one aspect of the case. This demonstrates how labeling can limit our perception and understanding of a situation.

There is an alternative path that the professional could choose. Although less intuitive, it is more complex and encapsulates the methodological component of the ARDESOS-DIAPROVE program. This sequential process requires the professional to recognize the general limitations that hinder correct or resolutive thinking, which could obscure the required decision making. However, it is not enough to just recognize these limitations; it is also crucial to identify them. In the program, this step is known as DEFISESGO, which refers to the identification of deficiencies and biases. By correctly applying this step, the professional would not focus solely on diagnoses and personality categorizations. Instead, she would analyze additional information, such as the sister’s behavior and business decisions after the incident. In this way, the representativeness bias is recognized and overcome, avoiding basing the analysis solely on the psychological profile, the police report, or the court decision.

In a later stage, the professional should explore alternative explanations that transcend the simple collection of third-party data. This process is called BUSEXPLICA (search for a unique explanation). This process is conceptually aligned with IME, at least in its first phase. In this process, the professional should explore alternative hypotheses that consider family and business dynamics to provide a better explanation of the observed facts and contribute to decision making. To accomplish this, she could meticulously examine all available data and evidence, including forensic reports, testimonies, and business records, and combine them with the data she has collected herself. However, one thing is to seek the explanation, and another very different thing is to obtain a single explanation verified by the judge, which is the facts. This last step is called MEXPLICA (to have the best explanation and seek to unequivocally explain a fact or problem). It is at this moment that the powerful but sometimes complex machinery of deduction (Govier 2010) and causality is set in motion. This last stage is associated with IBE by the categorization of unequivocal explanation, but above all, because there can be no doubt that it is this explanation and not another that should be the protagonist in decision making. With the data logically integrated, the professional has all the elements of judgment to make a decision based on the two hypotheses at stake. Hypothesis 1: the emotional instability of the new manager makes her incompetent to lead the company, backed by her mental health history, reasonable doubts about her responsibility in the death of her brother, and her behavior after the incident. Hypothesis 2: the new manager is able to lead the company, demonstrated by her ability to handle crisis situations, the forensic experts who exonerated her of any intentionality in the death of her brother, and her adaptability shown after the incident.

The ARDESOS-DIAPROVE program highlights in its analysis of the Lilly case, presented here in a summarized form, the relevance of basing explanations on evidence and observable facts. This approach underscores the need to overcome cognitive biases to facilitate decision making based on rigorous explanations derived from various sciences. The cases studied in the program are characterized by their ability to simulate reality, representing challenges that professionals must face. For this reason, the methodology focuses on the objective reality of the case, seeking to improve accuracy in the decision-making process. The application of the inference method to the best and only explanation, in this context, is especially effective. It facilitates the identification and support of the hypothesis that best aligns with the available evidence, leading to a more structured and logical analysis. This allows for a deeper and more precise understanding of the situation.

## 3. Conclusions

The aim of this article was to comprehensively expose one of the fundamental pillars of the ARDESOS-DIAPROVE program: inference to the best explanation. This aspect is crucial as it directly connects with the real world and refers to intriguing evidence that requires a cause to satisfy its explanatory character. The program focuses on the development of problem-solving skills through explanation. However, it is not about any explanation, but the best one. In this sense, it is essential to consider the reflections and challenges that the concept has faced in the epistemological approach.

An explanation is a powerful tool for understanding and solving problems, but only if it is accurate, relevant, and evidence-based. Unlike argumentation, explanation directly links to decision-making and real-world issues. Therefore, in teaching and practicing CT, it is essential not only to teach students to question and evaluate explanations but also to generate them effectively. For this, we take scientific activity as a reference. This involves understanding the criteria, methods, and scientific standards that allow obtaining the best explanation and, at the same time, being aware of the complexity and limitations of the explanation process itself. The ARDESOS-DIAPROVE program has solid foundations drawn from psychology and the most outstanding developments of the rich tradition of critical thinking and, above all, has incorporated successful pedagogical practices and strict compliance with scientific standards. Therefore, it is not surprising that, as a result of the dialogue between the epistemological approach and the program, there is a high degree of coincidence. To the extent that an educational intervention program incorporates scientific results and applies scientific methods in search of explaining and predicting reality in a rigorous, replicable, and objective way, the dialogue with any epistemological approach will be constructive.

With the Lilly case, we have tried to emphasize the importance of seeking reasonable explanations and grounded in concrete evidence to guide decision making. We certainly recognize that the nature of a good explanation varies according to context and discipline, indicating the need for a more detailed and contextualized analysis. In addition, CT, as manifested in this analysis, goes beyond simple first-order reasoning. It involves a deep and methodical reflection on one’s own thought process, including both the generation of conclusions through direct reasoning and the continuous evaluation of our cognitive abilities to overcome possible cognitive biases. The Lilly case demonstrates that an evidence-based and cognitive-bias-free approach, which prioritizes reasonable and contextualized explanations, is essential in knowledge processes and problem solving, but above all, it should guide decision making.

The ARDESOS-DIAPROVE program is an integrated system that relies on precise explanations to solve problems and substantiate informed decisions. The process begins with the identification and understanding of the problem. A misunderstanding at this stage can result in inadequate solutions or incorrect decisions. The program also promotes the creation of various explanations or hypotheses to discover viable solutions. This step requires creative exploration and logical reasoning. The proposed solutions undergo a critical analysis, considering factors such as coherence, applicability, and consistency with prior knowledge. Finally, the program highlights the need for rigorous evaluation to ensure that decisions are based on solid explanations. In summary, the program links decision making and explanation in a continuous cycle. Effective explanations arise from problem solving and guide future decisions. This process highlights the importance of critical thinking and the inference of the best and only explanation in the generation, evaluation, and selection of solutions, resulting in effective decisions and well-founded explanations.

Finally, epistemology, understood as a study of scientifically justified true beliefs, establishes conceptual spirals that reinforce scientific practice, especially highlighting methodological rigor. We have established dialogues within this explanatory spiral with selected authors. However, we could have extended these dialogues to other contemporary authors and established necessary contrasts, for example, with topics such as causality and probability or directly with abduction. These dialogues not only validate and conceptually strengthen the program but also allow us to participate in debates about these conceptual achievements. In recent years, we have not had the opportunity to participate in epistemological dialogues, mainly because the objectives of the program have been practical. We seek changes in students, their persistence over time, and their generality in reality. This sometimes leads us to assume practical commitments with applied research in CT without this being interpreted as a critical stance against the purely conceptual.

As has been demonstrated in this text, there are still many issues to debate, not only with this program but also with others. In addition, the door remains open to continue investigating the epistemological foundations of the proposals on CT and how they relate to the real world. An important direction for future research is how the role of CT, linked to epistemological debates, can mitigate cognitive biases in problem solving and decision making beyond the factual field of psychology. Perhaps the most relevant thing is how and under what conditions to measure the effectiveness of the program beyond a test that, although it can be predictive of behaviors in the world, is not the real world. These areas of research not only expand the scope of the current article but also open new avenues for understanding the practical application of critical thinking and epistemology in real life.

## Data Availability

Data are contained within the article.
